# Extracellular Vesicles in Oncology: from Immune Suppression to Immunotherapy

**DOI:** 10.1208/s12248-021-00554-4

**Published:** 2021-02-14

**Authors:** Akhil Srivastava, Shipra Rathore, Anupama Munshi, Rajagopal Ramesh

**Affiliations:** 1 Department of Pathology, University of Oklahoma Health Sciences Center, 975 N.E., 10th Street, Oklahoma City, Oklahoma 73104 USA; 2 Stephenson Cancer Center, University of Oklahoma Health Sciences Center, Oklahoma City, 73104 Oklahoma USA; 3 Department of Radiation Oncology, University of Oklahoma Health Sciences Center, 975 N.E., 10th Street, Oklahoma City, 73104 Oklahoma USA; 4 Graduate Program in Biomedical Sciences, University of Oklahoma Health Sciences Center, Oklahoma City, 73104 Oklahoma USA

**Keywords:** cancer, exosomes, liquid biopsy, immunotherapy, tumor microenvironment

## Abstract

Exosomes are involved in cell-to-cell communication and play a crucial role in cellular physiology. The role of exosomes in cancer has been widely explored. Tumor cells have evolved and adapted to evade the immune response. The study of the immune system’s modulations in favor of rogue tumor cells led to the development of a novel immunotherapeutic strategy targeting the immune checkpoint proteins (ICPs). In clinical settings, the response to ICP therapy has been inconsistent and is difficult to predict. Quantitating the targeted ICPs through immunohistochemistry is one approach, but is not pragmatic in a clinical setting and is often not sensitive. Examining the molecules present in bodily fluids to determine ICP treatment response, “liquid biopsy” is a convenient alternative. The term “liquid biopsy” refers to circulating tumor cells (CTCs), extracellular vesicles (EVs), non-coding (nc) RNA, circulating tumor DNA (ctDNA), circulating free DNA (cfDNA), etc. EVs includes exosomes, microvesicles, and oncosomes. Herein, we focus on exosomes isolated from bodily fluids and their use in liquid biopsy. Due to their unique ability to transfer bioactive molecules and perturb the physiology of recipient cells, exosomes have garnered attention for their immune modulation role and as a resource to identify molecules associated with liquid biopsy–based diagnostic methods. In this review, we examine the putative role of exosomes and their cargo in influencing the immune system. We discuss the immune and tumor cells present in the tumor microenvironment (TME), and the exosomes derived from these cells to understand how they participate in creating the immune-suppressive TME. Additionally, use of exosomes in liquid biopsy–based methods to measure the treatment response elicited by immunotherapy is discussed. Finally, we describe how exosomes have been used to develop immune therapies, especially cell-free vaccines, for cancer treatment.

## INTRODUCTION

Cancer is a unique pathological condition in which marked dysregulation of a series of molecular events and processes allows healthy cells to behave abnormally and, in many cases, aggressively. Since these changes are not caused by foreign molecules, it is interesting to observe how the body’s immune system, which is designed to eliminate such cancerous cells, is perturbed and allows these cells to go unchecked and display their effects by initiating cancer and its progression through metastases. Recent studies in the areas of immuno-oncology and the tumor microenvironment (TME) have revealed that immune cells and their soluble secretions bring about changes in the TME that allow cancer cells to establish and propagate (1,2). Research has confirmed the immune-suppressive activities of immunogenic cells, such as T cells, B cells, natural killer (NK) cells, and dendritic cells (DC), but the exact mechanism of their regulation is still unclear. However, a critical role of intercellular communication and signaling has been well established (3,4).

At a multicellular organizational level, the essential communication between cells is maintained through various mechanisms. One such mechanism is through tiny cellular vesicles that are collectively called extracellular vesicles (EVs). One subclass of EVs is exosomes. Exosomes are tiny, nano-sized (50 nm to 150 nm in diameter) vesicles produced and released by all cells through a well-defined and regulated endosomal pathway. Exosomes, due to their nature as cellular cargo carriers, were previously considered vesicles that removed toxic materials from the cellular lumen (5).

Although exosomes were discovered in the sixties, their functional importance was not recognized until recent. However, scientists recently discovered that exosomes carry several biologically active regulatory molecules, such as proteins, nucleic acids (DNA, several species of coding and non-coding long and small RNAs), and lipids, in their lipid bilayered membrane structure (5). The pivotal role of exosomes in cellular communication led to several studies and discoveries demonstrating exosomes as an important mediator in immunomodulation in the TME and their involvement in cancer pathophysiology (6–8). Immune cells and tumor cells in the TME secrete exosomes, which influence early and late events related to carcinogenesis. In 1996, the first evidence of the role of exosomes in immunogenicity was documented in B cell–derived exosomes that showed an immunomodulatory effect in major histocompatibility complex (MHC) class II restricted CD4^+^ T lymphocytes (9,10). Two years later, the Zitovgel group published their work showing the potential of DC-derived exosomes (DEX) in immunotherapy (11).

**Fig. 1:**
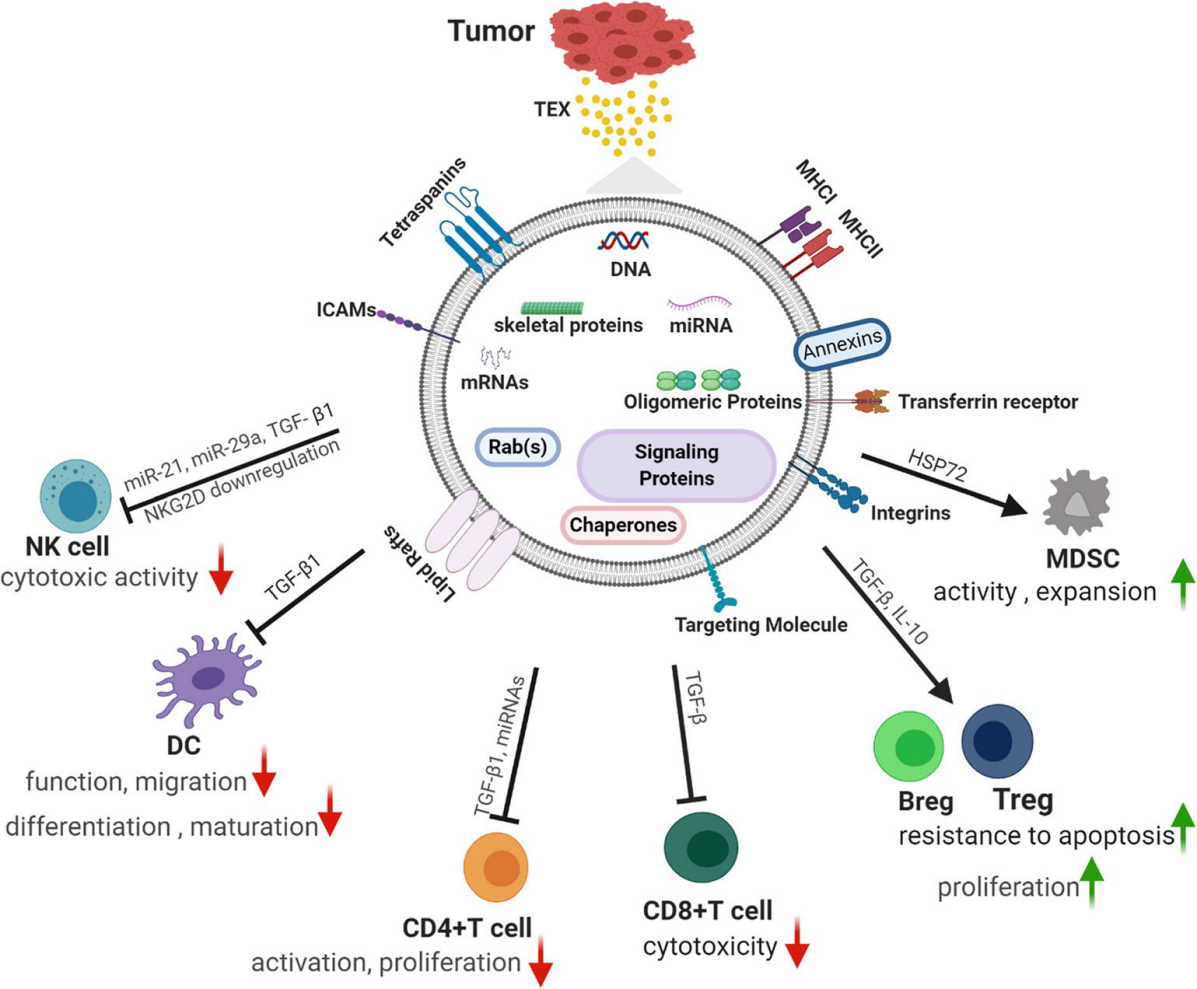
Exosomes from tumor cells (TEX) play an important role in modulating immune cells in the TME and promote different activities resulting in favorable condition for tumor propagation. Figure created with BioRender.com

Tumor cells are an important component of the TME. The exosomes released by tumor cells, hereafter referred to as “TEX” are enriched in molecules that are purportedly involved in immunosuppressive and inflammatory effects aiding in the development and establishment of favorable TME for tumor cells and metastatic cells (Fig. 1). DEX, B-cell, and macrophage-released exosomes promote the anti-tumor immune response (12,13). Thus, studying the exosomes produced by various cellular components present in the TME will enhance our understanding of cancer pathology and physiology, and aid in developing novel immunotherapeutic interventions. Immunotherapy has been successful in treating many cancers, including lung, breast, colon, cervix, head and neck cancer, and melanoma. Despite immunotherapy offering a new hope for cancer patients, especially those who have failed chemotherapy, treatment response has been variable when administered alone or in combination with conventional therapies.

Currently, the programmed death-ligand (PD-L1) levels estimated through immunohistochemistry (IHC) performed on tissue biopsy specimens is an accepted biomarker to predict the treatment response. However, conventional tissue biopsies are not sensitive enough. Since exosomes in the TME are involved in immunomodulation and can be isolated from different bodily fluids, use of exosomes in liquid biopsy–based diagnosis is a new strategy to develop predictive diagnostic biomarkers for immunotherapy.

This review describes the role of exosomes and their contents in immune stimulation, possible immunotherapeutic aspects, and their role in novel liquid biopsy modalities to develop sensitive and specific predictive biomarkers for a given immunotherapy. The use of exosomes in translational cancer research, their application in clinical trials, and various challenges associated with applying exosomes in cancer diagnosis and therapy are discussed herein. For more detailed information on exosomes influencing the immune response, readers are directed to read reviews by Yan *et al.* and Xie *et al.* (14,15).

## CONTENT OF EVS FOR IMMUNOMODULATION AND AS CANDIDATES OF LIQUID BIOPSY

The presence of molecular entities in the exosomes makes them unique in their functioning and significance. Studies have shown that the packaging of molecules into the lumen of exosomes is not a random process and occurs through a tightly orchestrated process during the biogenesis of exosomes in the late endosomal pathway. Exosome biogenesis begins with the formation of endosomes by endocytosis. As the early endosome transitions to late endosomes, the inner membrane starts folding or invaginating forming small vesicle-like structures inside the maturing endosomes. These small vesicles inside the endosomes are called intraluminal vesicles (ILVs) and the endosomes bearing them are called multi-vesicular bodies (MVBs). These MVBs are an important site for packaging of biomolecules into the ILVs that are released as exosomes when MVBs fuse with plasma membrane (16). During the process of MVB maturation and until ILVs are released, a battery of proteins under the umbrella of endosomal sorting complexes required for transport (ESCRT) play an important role in the packaging of proteins into the lumen of exosomes and release of exosomes from cells. This ESCRT machinery includes the ESCRT-0, ESCRT-I, ESCRT-II, and ESCRT-III protein complexes. In addition, the Rab proteins, which include Rab 27a, Rab27b, Rab35, and Rab11, are involved in the formation of ILVs and sorting of proteins and biological molecules (17–19). It is well established that the content of exosomes mirrors its cell of origin and hence one can examine the exosome content to determine its function and significance (20).

In the next sub-section, we describe important molecules present in the immunogenic exosomes and their putative functioning in cancer immunology. The exosomes derived from melanoma cells regulate the tumor immune response, mostly by instigating immune evasion by tumor cells. Hence, the exosomes and their contents can also be a therapeutic target (21). Using microscopy and flow cytometry techniques, CD8^+^ T cells were shown to internalize exosomes from different tumor types, even if these T cells do not internalize EVs as efficiently as do other immune cells. Further, the function of melanoma-derived exosomes in CD8^+^ T cells was examined. Researchers showed that these exosomes downregulated the T cell response through decreased T cell receptor (TCR) signaling and diminished cytokine and granzyme B secretions, which reduced the cells’ cytotoxic activity (22,23).

### Protein

In 1996, exosomes were first reported as nanovesicles that were enriched in major MHC-II molecules secreted from B-lymphoblastoid cells (9). This discovery led to the identification of exosome-like structures that were associated with all types of immunocytes, including B and T lymphocytes, macrophages, DCs, NK cells, mast cells, and thymocytes (10,24,25). Following the premise that exosomes play an integral role in immune modulation, the presence of numerous proteins derived from the immunocytes and enriched in the exosomes have been reported (26). In addition to cytokines, cytokine receptors, and integrin’s, the proteins that are present in the exosomes include adhesion molecules (CD11b, CD54/ICAM-1), antigen-presenting molecules (MHC class I, MHC class II, and CD1), T and B cell receptors, and costimulatory proteins (CD86) (10,27,28).

### Checkpoint Inhibitor as a Contributor to Liquid Biopsy

Immune checkpoint proteins (ICPs) function to restrict the immune system by providing inhibitory signals, and block the activation of T cells to enact self-tolerance (29). Tumor cells express these checkpoint proteins and successfully use them to evade the immune response (30). Novel immunotherapeutic interventions attempt to block these checkpoint proteins through antibodies. Higher expression of checkpoint proteins, such as programmed cell death-1 (PD-1), programmed death -ligand1 (PD-L1), cytotoxic T lymphocyte antigen-4 (CTLA-4), B7 homolog 4 protein (B7-H4), T cell membrane protein-3 (TIM-3), and lymphocyte activation gene 3 (LAG-3), has been observed in tumors. Based on the nature of exosome biogenesis, it is predicted that TEX should also have these checkpoint proteins present in their lumens and membrane surface (31–33). Chen *et al*. recently showed that PD-L1 is enriched in exosomes, when compared with that in melanoma microvesicles, suggesting that exosomes are the primary source of PD-L1 among EVs in this cancer type (34). Other studies have confirmed exosomal PD-L1 in melanoma, prostate cancer, breast cancer, glioblastoma, head and neck cancer, lung cancer, and other tumors (35,36). However, the success of immunotherapy depends on the expression profile of these different ICPs.

**Fig. 2:**
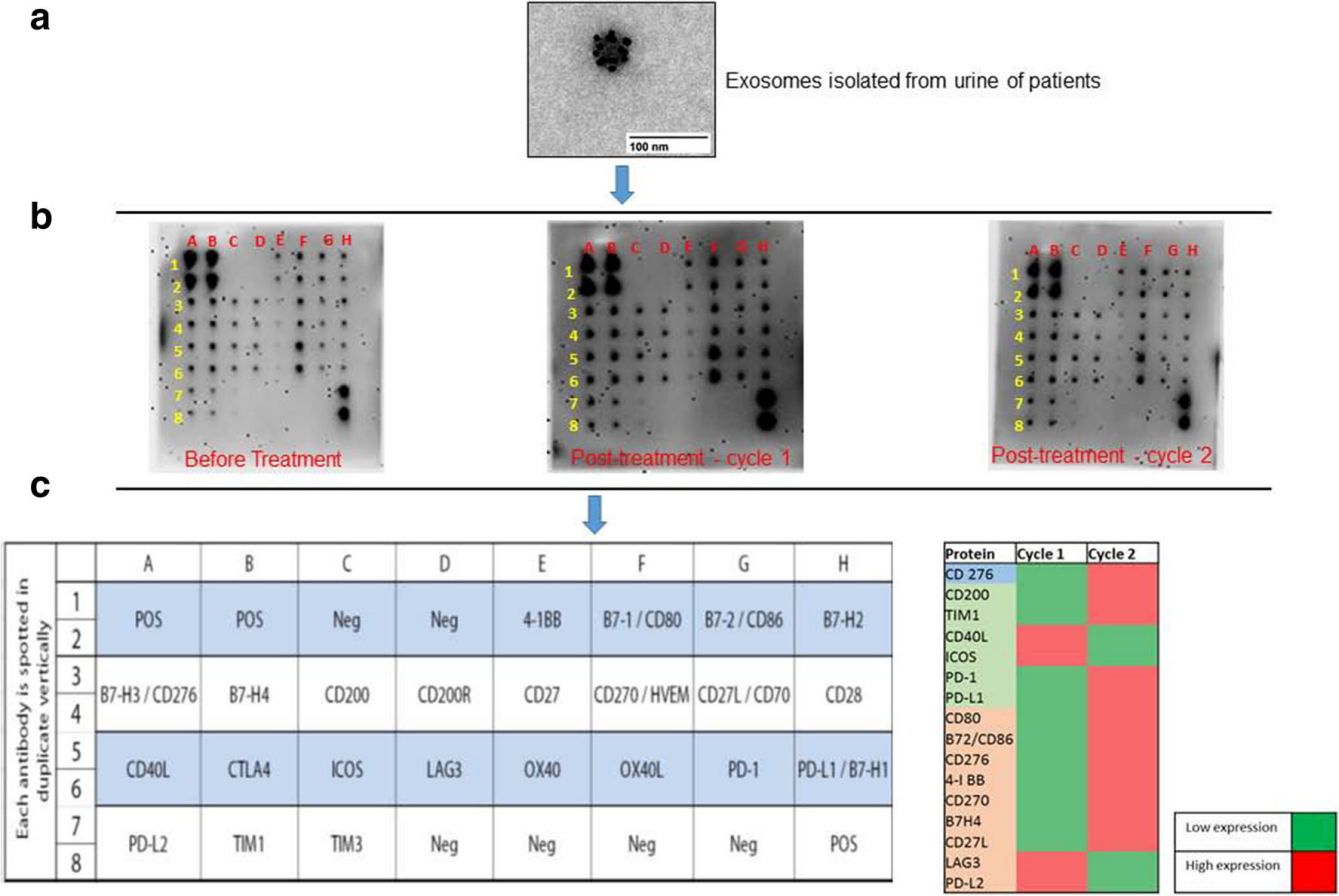
The exosomes contains various immunogenic proteins including immune checkpoint proteins. It has been hypothesized that these checkpoint proteins play crucial role in treatment outcomes. (**a**) Detection of PD-L1 in exosomes derived from the urine of non-small cell lung cancer (NSCLC) patients. Ant-PD-L1 antibody conjugated to gold was used to detect the presence of PD-L1 on the surface of exosomes. (**b**) A protein array detects twenty-three different immune checkpoint proteins on exosomes. (**c**) Expression analysis of immune checkpoint proteins show change in expression profile in response to the immune therapy

Liquid biopsy utilizes circulating tumor cells (CTCs), circulating tumor DNA (ctDNA), circulating cell-free DNA (cfDNA), micro (mi) RNA, and non-coding (nc) RNA for cancer diagnosis and in determining disease progression and treatment response. Exosomes isolated from bodily fluids carry the above-mentioned biomolecules. Moreover, exosomes have been successfully isolated in a non-invasive fashion from bodily fluids, such as urine and saliva. In our lab, we have explored the use of urine-derived exosomes from non-small cell lung cancer (NSCLC) patients receiving immunotherapy to develop a non-invasive liquid biopsy tool to predict treatment response. We successfully detected many ICPs in urinary exosomes from NSCLC patients treated with anti-PD-1 and chemotherapy (Fig. 2). We also observed a change in the ICP expression profile in response to the treatment, suggesting the putative role of exosomes in immune modulation and the possibility of testing their role as predictive markers for measuring therapeutic outcomes. Concurring with our findings, Cardonnier *et al*. (37) showed that in comparison to tissue biopsies, exosomal PD-L1 isolated from plasma of melanoma patients showed improved detection of disease incidence (67% in tissue biopsies *vs* 100% in blood plasma). Further this study also showed a correlation between exosome PD-L1 post treatment (ΔExoPD-L1) and tumor response to treatment with an 83% sensitivity, 70% specificity, 91% positive predictive value, and 54% negative predictive value for disease progression (37). Song *et al*. in their study speculated the probable mechanism of PD-L1 upregulation in exosomes in response to immunotherapy is by interferon-γ (IFN-γ) (38). Through mechanistic studies, the authors tried to explain melanoma patients who failed pembrolizumab therapy showed increased levels of exosomal PD-L1 post treatment. The study also established the role of exosome PD-L1 isolated from blood in stratifying patients as responders and non-responders indicating the utility of exosome PD-L1 as a treatment prediction marker (38). Gunasekaran *et al*. investigated the role of exosomal PD-L1 as a non-invasive tool for predicting the treatment response to immune checkpoint inhibitors on a small cohort (*n* = 25) of NSCLC patients (39).

Apart from PD-L1, PD-L2 expression on exosomes has also been reported (40). Examination of exosomes from IL-10 treated murine dendritic cells showed reduction in exosomal PD-L1 and PD-L2 did not contribute to the immune-suppressive function in a murine model of sepsis (40). It is evident from this study that exosomal PD-L2 could also serve as potential biomarker in cancer diagnosis and therapy. Finally, testing of additional ICP proteins on exosomes as predictive liquid biopsy biomarker in cancer patients receiving various ICP inhibitors would improve understanding the molecular mechanisms of resistance and failure to immunotherapy (41,42).

### Lipids

In addition to unique sets of proteins, the lipid composition of exosomes is distinct from other cellular membranes. Exosomes are enriched in membrane lipids and are deprived of cytosolic lipids. More than 2000 lipid molecules have been described to be present in the exosomes (43). Sphingomyelin, glycosphingolipids, phosphatidylserine, and cholesterol are enriched two to three times in exosomes compared to cells. Exosomes have no transmembrane phospholipids present in them. In contrast, phosphatidylcholine and phosphatidylinositol are more enriched in cells compared to exosomes (44). Lipidomics study of immunocytes like B cells and mast cells showed the presence of cholesterol (45). The high content of lipids in the exosome membrane is implicated in facilitating the interaction between exosomes and cells (46,47). Like nucleic acids and proteins, packaging of lipids into exosomes occurs through a highly regulated process and often reflects the state of cells. Several studies have explored the possibility of using lipid profiles for the identification of putative biomarkers. Skotland *et al*. (46) identified one hundred and seven lipid species in the exosomes of urine samples collected from prostate cancer patients and healthy controls. In this study, the authors identified nine lipid species that showed significant differences in the urinary exosomes of prostate cancer patients compared to normal individuals. Phosphatidylserine (PS) 18:1/18:1 and lactosylceramide (d18:1/16:0) showed the highest patient- to-control ratio with 93% sensitivity and 100% specificity (46). Brzozowski *et al.* profiled lipidomics on exosomes from non-tumorigenic (RWPE1), tumorigenic (NB26), and metastatic (PC-3) prostate cancer cell lines. They identified 187 unique signatures in lipid profiles and identified fatty acids, glycerolipids, prenol lipids, sterol lipids, sphingolipids, and glycerophospholipids as candidate biomarkers (48). As a unique strategy of harnessing lipid profiling as biomarkers, Cheng *et al*. combined the proteomic and lipidomics of exosomes isolated from ovarian cancer cell lines and ovarian surface epithelial cells. They identified 1433 proteins and 1227 lipids in the exosomes derived from the two cell lines and observed significant differences in several lipids and proteins in the two exosomes populations. Their study implied combination of proteomic and lipidomic profiles could be a promising approach in the early diagnosis of ovarian cancer (49). Application of such unique approaches make exosomes excellent candidates for developing biomarkers and strategies for liquid biopsies.

### Nucleic Acids

Different species of nucleic acids (gDNA, miRNA, lncRNA, mRNA, etc.) have been identified in tumor-derived exosomes (TEXs). Several non-coding small RNAs, such as miRNAs, are present in high levels in the exosomes and are considered potential circulating diagnostic biomarkers for many cancer types, such as glioblastoma, melanoma, and prostate cancer, and thus forms a major contributor of liquid biopsies (50–52). The presence of coding mRNAs and an array of non-coding small RNAs, such as Piwi-interacting RNA (piRNA), small non-coding RNA (sncRNA), short interfering RNA (siRNA), and miRNAs, have also been reported (53). Additionally, the presence of DNA, including single-stranded (ss) and double-stranded (ds) DNA, genomic (g) DNA, and even mitochondrial (mt) DNA, in the exosomes have been reported (54). However, the presence of gDNA in the exosomes is under debate due to conflicting results presented warranting additional studies with thorough investigation (55). The presence of nucleic acids in the exosomes has led to their investigation in immunosuppression during tumorigenesis, molecular cues in generating an immunocompromised TME, and favorable metastatic niche. In the following sub-sections, different nucleic acid molecules present in the exosomes and their role in immuno-oncology are discussed.

#### miRNAs

miRNAs are small 22–24 nucleotide non-coding RNAs that regulate gene expression and are present in cells and body fluids, and constitute a major component of the exosomal cargo. miRNAs packaged in the membrane-encapsulated vesicles are protected from RNA-digesting enzymes and participate in several regulatory functions in the recipient cells (56,57). The packaging of miRNAs into the exosomes occurs through a well-regulated mechanism. Research has shown that certain guiding sequences dictate the loading of miRNAs into the exosomal lumen (58). Additionally, the putative role of some RNA binding proteins in packaging the miRNAs into exosomes is currently under investigation.

miRNAs are functionally important species among all other molecules present in the exosome cargo (59). Hence, exosomal miRNAs are important in imparting immune evasion by tumor cells. The miRNAs from exosomes can stage this immune evasion in several ways, including immune cell modulation and altering the immune response. Further, exosomes derived from several immune cell types contain miRNAs to perform the function of immune modulation in the TME (23,60). The exosomal miRNAs in addition to influencing the oncogenic behavior of tumor cells can serve as molecular targets for cancer therapeutics; in the identification of new diagnostic biomarkers; and in the assessment of treatment response. Table I lists various miRNAs isolated from different TEX and their influence on the immune system during cancer pathogenesis.

**Table I:** Exosome-Derived miRNAs in Tumor Immunology

miRNA ID	Exosome origin	Functional role	Reference
(hsa)-miR-3187-3p, hsa-miR-498, hsa-miR-122, hsa-miR149, and hsa-miR-181a/b	Melanoma	Downregulate CD 8^+^ T cell response by decreasing T cell receptor (TCR) signaling and TNF-α secretion	(23)
miR-21 and miR-29a	Tumor cell	Bind to Toll-like receptors (TLRs) expressed on macrophages, and stimulation of NF-κB-mediated secretion of cytokines to produce a pro-inflammatory TME	(61)
miR-223	Tumor-associated macrophages (TAMs)	Development of aggressive and invasive phenotypes in breast cancer.	(62)
miR-23a	Tumor cell	Interference with the functioning of NK cells	(63)
miR212-3p	Pancreatic cancer	Target MHC class II promoting immune evasion of tumor cells	(63,64)
miR-203	Pancreatic cancer	Regulate TLR4 to influence the functioning of DCs	(65)
miR-24-3p	Nasopharyngeal cancer	Impaired T cell proliferation and differentiation to Th17 cells.	(66)
miR-152	Non-small cell lung cancer	Regulates fibroblast growth factor (FGF)2, affecting the cell growth and migration	(67)
miR-24-3p, miR-891a, miR-106a-5p, miR-20a-5p, and miR-1908	Nasopharyngeal cancer	Development of tumor immune-suppressive TME by promoting regulatory T cells (Tregs)	(68)
miR-92b	Hepatocellular carcinoma	Suppress CD69 expression and NK cell-mediated cytotoxicity	(69)
miR-222-3p	Epithelial ovarian cancer	Suppress cytokine signaling (SOCS3), polarize macrophage to M2 phenotype, and create an immunosuppressive niche for tumor development and metastasis	(70)
miR-let-7a-5p, miR-10a-5p, miR-1246, and miR-125b-5p	Lung adenocarcinoma	Promote polarization of macrophages (M1 to M2) to create an immunosuppressive pro-inflammatory environment.	(6,69)
miR-210	Lung cancer	Develop immunosuppressive TME	(68)
miR-155 and miR-125b-2	Pancreatic cancer	Promote polarization of macrophages (M1 to M2) to create an immunosuppressive pro-inflammatory environment	(71,72)
miR-214	Lewis lung carcinoma	Promote Tregs cells	(71,73)
miR-940	Epithelial ovarian cancer	Activate M2 phenotype to create an immunosuppressive pro-inflammatory environment	(74)
miR-301a-3p	Pancreatic cancer	Activate PTEN to trigger M2 polarization of TAMs	(6)
miR-24-3p	Tumor cell	Block T cell proliferation to induce immune suppression via regulating the ERK- and STAT-signaling pathways	(66)
miR-1246	Colon cancer	Reprogram macrophages into a tumor-supporting state.	(75)
miR-92a	Leukemia	Pro-tumor TME and immune evasion	(76)
miR-142-5p	Pancreatic cancer	Increase cytotoxic CD4^+^ and CD8^+^ T cells	(77)

#### DNAs

The presence of DNA in the exosome cargo has underscored the importance of DNA in exosome physiology (78,79). However, there are also studies that challenge the existence of DNA in exosome lumen (55). The functional significance of exosomal DNA (exoDNA) compared with exosomal RNA has not been widely studied. Nevertheless, exoDNA is involved in transmitting crucial genetic information from the donor cell to recipient cells, bringing about changes, including immunogenic changes, in the recipient cells. Some studies have provided insights into its putative role in immune cell response. The mechanism of DNA packaging to exosomes is unknown, but initial experiments on astrocytes and glioblastoma cells by Guescini *et al*. indicate the presence of mitochondrial DNA (mtDNA) in exosomes (80). Thakur *et al*. showed that TEXs contain exoDNA, the constituents of which were similar to those of the cell (cancer cell) of origin (81). Similar observations were reported in lung, breast, melanoma, pancreatic, and prostate cancer. *In vitro* and *in vivo* studies in glioblastoma and medulloblastoma cells demonstrated the presence of single-stranded DNA (ssDNA), gDNA, complementary (c) DNA, and transposon DNA (82,83,84). Two studies reported that exoDNA can play a pivotal role in modulating innate immune response. Lian *et al*. (85) and Kitai *et al.* (86) showed that cells under stress tend to produce more exoDNA by activation of DCs through cGAS-STING and the AIM-2 cell signaling pathway. These findings show that in addition to RNAs, DNA can have functional significance in regulating the immune system and could be novel immunotherapy targets.

## Exosomes in Immune Suppression Through Modulation of Immunocytes

The TME is a dynamic region involved in the initiation of malignant lesions and creation and establishment of the metastatic niche. Exosomes that are derived from antigen-presenting cells (APCs) and other immunocytes can present antigens to T cells and activate immune responses *via* classical MHC-restricted mechanisms, involving multiple cell–cell interactions (15,87–89). Exosomes function in initial T cell priming, differentiation of mature T cells, and in the development of effector functions. Antigen presentation by exosomes can be accomplished by transfer of MHC peptide complexes between naïve DCs, uptake and presentation of exosome cargo by mature DCs, and direct T cell activation without the need for uptake and further processing by APCs (10,90,91). Immunogenic cells, such as CD4^+^ T, CD8^+^ T, regulatory T cells (Tregs), NK cells, and DCs, are populated in TMEs. These cells and their respective exosomes have demonstrated key functions in stimulating or attenuating immune responses that rely on the functional molecules in the cargo and the microenvironment in which the exosomes are produced.

DCs are the major type of APCs involved in both innate and adaptive immunity. In cancer pathophysiology, DC-derived exosomes promote and oppose oncogenic activities through two subtypes: exosomes from mature DCs (mDex) and exosomes from immature DCs (imDex). Researchers have shown high levels of MHC class II and other ICP proteins and reduced levels of milk fat globule EGF factor 8 (MFG-E8) in exosomes from imDex result in antigen-specific T cell proliferation and *in vivo* immune responses. Under malignant conditions, the TEXs perturb the maturation and differentiation of DCs (28,92,93). TEXs modulate the functioning of DCs through the presence of heat shock protein (HSP)72 and HSP105 proteins on their surfaces, inducing interleukin (IL)-6 secretion, which culminates in tumor metastases (94). TEXs can inhibit both differentiation and maturation of DCs. In pancreatic adenocarcinoma (PDAC), tumorigenesis is promoted by suppression of the function of DCs by exosomes carrying miR-203, which downregulates Toll-like receptor 4 (TLR4), tumor necrosis factor-α (TNF-α), and IL-12 expression, and ultimately prevents antigen presentation by DCs (95). In a similar fashion, exosomes enriched in miR-212-3p and isolated from pancreatic cancer cells when taken up by DC cells downregulate the regulatory factor X-associated protein (RFXAP) expression, which subsequently decreases MHC class II expression and results in the inactivation of CD4^+^ T cells, contributing to the generation of an immunotolerant microenvironment in PDAC (64). Therefore, studies suggest that targeting the release of miR-203- and miR-212-3p-positive TEXs could result in positive treatment outcomes in PDAC patients (64,95).

TMEs are enriched in Tregs that create an immunosuppressive environment to promote oncogenic properties. Treg-derived exosomes have also been shown to maintain immune tolerance (96). Interaction of DCs with exosomes produced by Tregs and transfer of miRNA-150-5p and miR-142-3p from the Treg exosomes to DCs suppresses DCs functions and leads to an immunosuppressive TME (97).

Macrophages are another class of tumor-infiltrating immunocytes that are abundant in the TME. These cells are designed to engulf and destroy bodies of foreign origin or those that work against the normal physiology of the cell. Malignant tumors hijack this potential of macrophages in the TME and re-engineer them as tumor-associated macrophages (TAMs), which are then exploited by tumors for sustainability and successful proliferation. Macrophages can change polarization from conventional pro-inflammatory (M1) to immunosuppressive (M2) cells depending on the physiological conditions. Studies have revealed that functional differences in M1 and M2 cells are attributed to their molecular contents. M1 cells express stimulatory molecules such as CD80, CD86, iNOS, TLR2, TLR4, cytokines (IFN-γ, TNF-α, IL-12, IL-23), and chemokines (CCL5, CCL9, CCL10). M2 cells express high levels of CD163, CD23, CD204, CD206, VEGF, and arginase (Arg)1, in addition to cytokines like IL-10, TGF-β, TGF-α, TNF, and chemokines such as CCL16, CCL17, and CCL22. The transition from M1 to M2 is very dynamic in nature and regulated by cellular signaling pathways in the TME. In a recent review, Cheng *et al*. have elegantly discussed the differentiation of M1 to M2 macrophages and their regulation and implications in cancer (98). Recent evidence from several studies has established that exosomes are intimately involved in the various cellular signaling mechanisms; hence, it becomes obvious that they will have important role in the polarization of macrophages under specific physiological conditions, such as cancer. A review by Baig *et al*. extensively discuss the role of TEXs in macrophage polarization and differentiation (99).

Conventionally, M2 macrophages promote pro-oncogenic activities, anti-inflammatory responses, and metastases. TAMs are largely regarded as M2 macrophages. The presence of M2 macrophages has been reported in many invasive cancers (e.g., pancreatic cancer, brain cancer), contributes to tumor progression by lymphangiogenesis, and correlates with lymphatic metastases and poor survival (100–103). Chemokines and other molecular cues in the TME are considered responsible for TAMs/M2 phenotypes’ appearance. Recently, exosomes’ role in TAM/ M2 polarization and its underlying effect on metastasis and other cancer-related activities have been discussed. Tumor cells produce a hypoxic microenvironment, and the TEXs produced under hypoxia can polarize macrophages to the M2 phenotype in a HIF-1α- or HIF-2α-dependent manner (104). In ovarian cancer, TAM/M2-derived exosomes have been shown to carry and deliver miRNAs that induced a Treg/Th17 cell imbalance to promote disease development (105). It is also reported that exosomes can activate T cells such as CD8^+^ and CD3^+^ cells. Consequently, exosomes produced from these activated T cells participate in IL-2-mediated immune response (106,107). Exosomes produced from the activated T cells interestingly are large and is presumably due to the packaging of a large number of cytokine molecules. These studies suggest that immunocyte-derived exosomes might have both beneficial and deleterious effects on the immune response and their functions dictated by the *in situ* TME (108).

T cell–derived exosomes are known to be involved in the invasion and metastasis of different cancers. As an example, exosomes expressing FasL isolated from activated CD8^+^ T cells though did not show any effect on apoptosis and proliferation of tumor cells promoted the invasion of B16 melanoma and 3LL lung cancer cells via the Fas/FasL pathway (109).

All of these study results demonstrate exosomes from immune cells present within the TME contribute in the remodeling of the tumor stroma, specifically by establishing an immunosuppressive microenvironment.

## EVs IN IMMUNOTHERAPY 

Apart from studies investigating exosomes in immune modulation and their contribution in reconfiguring TME and promoting tumorigenesis, the use of exosomes as drug carriers for cancer therapy has also been examined (110). The exosomes due to their small size (50–150 nm) and being non-immunogenic due to their bodily origin make them an ideal alternative as drug carriers in cancer therapeutics (111–114). Further, the endogenous origin of exosomes and the ability to load desired molecules into their lumen (exogenously) has opened avenues for the use of exosomes as nanocarriers for targeted delivery of tumor-specific antigens and immunomodulatory drugs for cancer treatment (5,115). Preclinical studies demonstrate exosomes derived from normal cells (e.g., fibroblasts, DCs, macrophages, mesenchymal stem cells) can be designed to operate as carriers of anticancer drugs, imaging agents, and their combination to serve as a theranostic (116,117). Additionally, attachment of various tumor-targeted ligands on the exosomes surface resulted in tumor-targeted drug delivery and anticancer activity both *in vitro* and *in vivo* (118,119). The results from these studies demonstrate exosomes are stable and are not susceptible to physical and chemical manipulation. An added advantage of using exosomes as drug carriers is that they can avoid phagocytosis by circulating macrophages and monocytes via CD47 (120). Finally, exosome derived from immune cells (DCs, T cells, macrophages) have been used as cancer vaccines and for immunotherapy (121,122). Studies show that these exosomes elicit robust immune response independent of MHC molecules (123). Additionally, no marked therapeutic benefits were observed when exosomes from autologous cells were used and compared to exosomes from allogeneic cells (124). These observations open avenues for testing allogeneic cell–derived exosomes in immunotherapy and vaccine-based cancer therapies. Srivastava *et al.* have extensively described several strategies for engineering parental cells for increased exosome production and loading the exosomes with anticancer drugs and immunotherapeutics for use as nanocarriers for cancer treatment (110). The use of exosomes for combined delivery of immunomodulatory agents and chemotherapy could be another approach to develop a potent therapeutic intervention with minimal side effects. Currently, there are several phase1/2 clinical trials testing exosomes as cancer vaccine, combinatorial therapy, and as a source of biomarkers to monitor and predict disease progression and treatment response (Table II). Thus, exosomes hold promise in the areas of cancer diagnosis and treatment.

**Table II:** List of Exosomes and Exosome-Based Liquid Biopsy Clinical Trials (CT) in Immune Oncology (www.clinicaltrials.gov)

NCT number	Title	Status	Conditions	Interventions	Phase
NCT01159288	Trial of a Vaccination With Tumor Antigen-loaded Dendritic Cell-derived Exosomes	Completed	Non-small cell lung cancer (NSCLC)	Sequential treatment of chemotherapeutic agent; metronomic cyclophosphamide (mCTX) or mCTX and immunotherapy using antigen-loaded dendritic cell-derived exosomes (Dex).	Phase 2
NCT04427475	Prediction of Immunotherapeutic Effect of Advanced Non-small Cell Lung Cancer	Recruiting	NSCLC	This CT explores response of immunotherapy (pablolizumab and nafulizumab) by measuring the PD-L1 and miRNA levels in plasma exosomes in pre- and post-treated patients and correlate it with the treatment outcome.	Not applicable
NCT02869685	Clinical Research for the Consistency Analysis of PD-L1 in Lung Cancer Tissue and Plasma Exosome Before and After Radiotherapy	Unknown status	NSCLC	This CT is based on the premise that radiation therapy can enhance the effect of immunotherapy. The study involves measuring PD-L1 expression levels in tissues and plasma exosomes (pExo) of patients before and after radiation treatment.	Not applicable
NCT02890849	Clinical Research for the Consistency Analysis of PD-L1 in Cancer Tissue and Plasma Exosome	Unknown status	NSCLC	This CT tends to explore the consistency of PD-L1 levels in cancer tissues and plasma exosomes (pExo) so that pExo can be utilized as a modality to monitor response of radiotherapy and immunotherapy.	Not applicable
NCT02507583	Antisense102: Pilot Immunotherapy for Newly Diagnosed Malignant Glioma	Active, not recruiting	Malignant glioma	This CT uses the unique property of exosomes where they carry tumor antigens. The investigators of the study planted exosomes along with an anti-	Phase 1
NCT02439008	Early Biomarkers of Tumor Response in High Dose Hypofractionated Radiotherapy Immune Response	Terminated	Hepatocellular carcinoma, colorectal neoplasms melanoma kidney neoplasms	To use blood plasma derived exosomes (nanovesicles) collected before, during, and after radiation therapy to determine the treatment response and develop a liquid biopsy modality	Not applicable
NCT01550523	Pilot Immunotherapy Trial for Recurrent Malignant Gliomas	Completed	Malignant glioma of the brain	This CT is based on similar premise of NCT02507583 described above. The modification included in this study is use of a new oligodeoxynucleotide sequence and treatment at initial diagnosis	Phase 1
NCT03985696	Exosomes and Immunotherapy in Non-Hodgkin B-cell Lymphomas	Recruiting	Lymphoma, B cell, aggressive non-Hodgkin (B-NHL)	This CT explores the role of exosomes in immunotherapy escape by investigating therapeutic targets CD20, and PDL-1 carried by exosomes derived from B cell non-Hodgkin lymphoma (B-NHL) cells. The CT also evaluates exosome composition for identification of potential diagnostic markers	Not applicable
NCT03854032	Nivolumab and BMS986205 in Treating Patients With Stage II-IV Squamous Cell Cancer of the Head and Neck	Recruiting	oral cavity, larynx, hypopharynx, nasal cavity/paranasal sinuses, stage 1 oropharyngeal with lymphadenopathy	This CT basically explores the therapeutic efficacy of nivolumab and BMS986205 in combination or as standalone therapy. Exosome content and the number of exosomes present in peripheral blood will be used to assess the interactions between the immune and metabolic microenvironment. The exosomes will be isolated before, during, and after treatment.	Phase 2

## EV-BASED LIQUID BIOPSY TOOL FOR CANCER

In the previous section, we noted that exosomes could be used to improve the efficacy of immune therapeutics. The success of these immunotherapies depends on the ability of cancer cells to express a number of receptor proteins and ligands on their surfaces. These proteins can be used as targeting moieties or therapeutic targets, and feasibility studies to quantify these molecules’ expression can tremendously aid in enhancing the efficacy of immunotherapy. Based on this premise, researchers have worked to develop tissue- and cell-free or exosome-based liquid biopsies. Exosomes can be conveniently isolated from bodily fluids, such as blood (minimally invasive) and urine (non-invasive), and reflect the status of the cell of origin. Thus, investigating the content of exosomes is expected to provide specific information about critical components, including miRNA levels, ICP levels, and tumor mutational burden, which can be utilized as a prognostic or predictive biomarker for immunotherapy.

Additionally, exosomes offer many advantages to be explored for biomarkers and liquid biopsies. Several studies have shown exosomes are very stable and can be stored at − 80°C for a very long period of time, translating that the unique signature molecules carried by them are also stable and can be studied over time (125,126). The extraordinary stability bestowed to biomolecules enclosed in the lumen of exosomes also enhances the accuracy in those molecules’ diagnostic potential increasing their utility in liquid biopsies. cfDNA and CTCs are highly unstable and difficult to isolate from body fluids. In contrast, exosomes can be isolated in large quantities from body fluids like blood serum, cerebrospinal fluids, and urine. Finally, exosomes also show uniqueness as they, as a single particle, can simultaneously carry multiple biomolecules such as miRNA, lncRNA, mRNA, DNAs, lipids, cytokines, and proteins allowing investigation of more than one molecule as a candidate biomarker. This property will further enhance their precision and rigor in diagnosis or predicting treatment response. In thoracic malignancies and several other cancers, exosome-based liquid biopsy predictive biomarkers have already been described (127,128). In a recent study, Chen *et al*. discovered PD-L1 is packaged in the lumen of exosomes, and the levels of PD-L1 in exosomes played a crucial role in dictating the treatment response of anti-PD-1 antibody pembrolizumab immunotherapy in patients diagnosed with metastatic melanoma (34). The general liquid biopsy approach utilizes invasive and non-invasive or minimally invasive methods. The invasive methods primarily included plasma- and serum-derived exosomes to explore the encapsulated molecules for candidate biomarkers; however, some other bodily fluids have also been studied to identify biomarkers. In a recent study, Rodríguez *et al*. demonstrated that the quantity of exosomes was significantly increased in the bronchoalveolar lavage samples of cancer patients compared to that of non-cancer subjects (129). Pleural effusion is also a rich source of liquid biopsies. Lin *et al*. explored pleural effusion–derived exosome miRNA in pneumonia and lung cancer. The study discovered 27 differentially expressed miRNAs among which the miR-205-5p and miR-200b were significantly upregulated in lung cancer, suggesting that these two miRNAs could be used for distinguishing lung cancer from pneumonia (130). In another study Hydbring *et al*. evaluated pleural effusions from 18 adenocarcinoma patients and 18 with a benign inflammatory condition. They compared the exosomal miRNA profile between the groups and found miR-200 as a putative candidate for diagnosis of adenocarcinoma (131).

The second category of liquid biopsy is non-invasive methods consisting of bodily fluids such as urine, saliva, and breast milk. Li *et al*. examined the diagnostic potential of urine-derived exosomes in the diagnosis of NSCLC. The study revealed that in urinary exosomes, leucine-rich α-2-glycoprotein (LRG1) was expressed at higher levels as compared to matched cancer-free control indicating the potential use of urine-based exosomal diagnostic biomarkers (132). Studies conducted in our laboratory also demonstrated the diagnostic potential of miR-200c, which was enriched in the urine-derived exosomes of endometrial cancer patients compared to asymptomatic normal individuals (114). Urine thus has proven to be a useful resource for developing exosome-based liquid biopsy modalities for bladder, renal, and prostate cancers. The exosome content, especially proteins and miRNAs, has been extensively studied to identify putative diagnostic or prognostic biomarkers for bladder cancer. Proteins alpha-1 antitrypsin (SERPINA1) and histone H2B type 1-K (H2B1K) have been identified from urinary exosomes as promising biomarkers for prognosis in bladder cancer (133). Similarly, miRNAs like miR-21, miR-93, miR-200c, and miR-940 present in urine-derived exosomes were identified as a molecular signature for urothelial carcinoma of the bladder (UCB). The high degree of specificity determined in this study holds urinary exosome miRNA as a strong contender for developing a promising clinical diagnostic tool (134). Clinical assessment of prostate cancer through urine-derived exosomes has also been investigated in numerous studies (135–138). Similarly, in renal carcinoma, mass spectrometry analysis of urine-derived exosomes have helped in the identification of multiple proteins as candidate biomarkers (139). Human breast milk is a rich source of exosomes representing the status of tissues of the breast. Thus, the probability of obtaining breast-specific exosomes is much greater in human milk than in serum, blood, or urine. Biomarkers for several breast-related ailments can be investigated in breast milk and develop as a liquid biopsy modality (56). Exosomes from breast milk collected from involuting breast have shown to carry proteins that have pro-oncogenic effects. Matrix metalloproteinase 2 (MMP-2), MMP-3, and MMP-9, which are involved in reorganizing breast tissues to the pre-pregnancy stage, have also established their role in promoting breast metastasis (140). Transforming growth factor beta (TGFβ) isoforms TGFβ1 and TGFβ2 are also enriched in breast milk–derived exosomes and involved in tumorigenesis (141).

In addition to proteins, Sauter *et al*. has also discussed the differentially enriched miRNAs in breast milk and their putative role in breast cancer (140). Although these molecules have been investigated for their influence in the pathophysiology of breast cancer, in addition, their enrichment under a specific condition and its correlation with the development of cancer can be explored for the discovery of predictive biomarker (142).

Saliva has long been traditionally studied for tumor biomarkers for oropharyngeal cancer (OPC) and human papilloma virus (HPV) associated OPC. Wang *et al.* explored saliva exosomes for the identification of candidate biomarkers. Exosomes from saliva were isolated using an acoustofluidic platform and investigated for small RNA and DNA content using droplet digital RT-PCR. The study demonstrated the enrichment of HPV16 DNA in the exosomes isolated from the saliva of HPV-OPC patients suggesting their putative application in saliva exosome–based liquid biopsies (143). In their review article, Nonaka and Wong have discussed in detail the current status of salivary exosomes and their molecular content in the context of developing diagnostic and prognostic biomarkers and developing saliva exosomes as a novel modality of liquid biopsies. This context in the article has coined the term “Saliva-Exosomics” (144). We strongly recommend readers to refer the article to assess the potential of saliva exosomes in developing biomarkers and their application in cancers.

Despite the availability of many novel avenues, tissue biopsies are still the gold standard for cancer diagnosis. Nevertheless, it is not a perfect method as the issues with sensitivity in tissue biopsy are still lingering. In addition to the tedious process of obtaining tissue specimens, its storage and transportation are peripheral issues that make this process cumbersome. As mentioned before, exosomes are directly produced from the tumor cells and are present in all bodily fluids. Hence, their efficient isolation and follow-up analysis bring them to the forefront of diagnostic modalities. The current attention that exosomes receive from the scientific community will undoubtedly open new avenues to develop, test, and establish exosome-based liquid biopsy methods for diagnosis and assess the treatment outcome in the near future.

## FUTURE ROLE

In the sections above, we described the tremendous progress made in the field of EVs, in particular on exosomes and their application in liquid biopsy as a source of biomarkers for cancer diagnosis and as a drug delivery vehicle for cancer therapy. We have also seen how assessment through liquid biopsy presents many advantages, such as sample collection through a non-invasive or minimally invasive procedure (145,146). In addition, since exosome-based liquid biopsies rely on proteomic or genomic analysis, availability of a robust automated protocol will make the process of diagnosis fast and convenient. However, several challenges in advancing exosomes as a diagnostic tool and drug carrier exists. In the area of diagnosis, the method of isolation and characterization of exosomes needs to be addressed. It is difficult to discriminate TEXs from exosomes derived from normal cells when both populations are mixed and circulating in bodily fluids. While antibody capture method that relies on expression of specific antigen on TEXs has been developed, inconsistencies in the expression levels of the antigens on the exosomes have hindered advancement of this technology. Exosomes like CSCs continue to present varying antigens on their surface and hence pose challenge in reproducibility. Another area that need to be addressed for ensuring rigor and reproducibility is in defining exosomes quantity. Majority of the studies report exosomes quantity in terms of protein concentration and is per recommendation by ISEV. However, for clinical use in particular in the area of drug delivery, it is impractical to express exosome quantity in terms of protein concentration. Rather, it will need to be expressed in terms of particles per milliliter akin to the expression of viral particles used in cancer gene therapy studies. This approach will ensure rigor and reproducibility among different laboratories and clinical studies. Third concern that needs to be addressed is whether to use exosomes from autologous cells or from allogeneic cells for cancer vaccine and drug delivery as concerns of cross-reactivity and eliciting deleterious immune response exist. Use of autologous cells is beneficial for personalized medicine and recognition of specific tumor antigens. However, scaling-up of exosomes from autologous cells in particular if it has to be from individual patients could be time consuming. In contrast, use of exosomes from allogeneic cells can be easily scaled-up and in fact, better antigen presentation and cross-antigen presentation can occur resulting in enhanced immune response. However, detailed studies comparing exosomes from allogeneic and autologous cells are warranted. Improvisation in exosome production in large scale is another challenge to be overcome for clinical translation (147,148). Finally, since exosomes are heterogeneous in size (50–150 nm), it will be of interest to study if size matters for drug delivery and anticancer activity. This is especially important, as size and shape of various nanomaterials have been shown to impact half-life, cell uptake, biodistribution, and efficacy. Continued advances made in the biology of exosomes and technology development should result in overcoming the current limitations and result in the broad use of exosomes in clinical applications in the next few years.

## References

[1] Arneth B. Tumor microenvironment. Medicina (Kaunas). 2019;56(1). 10.3390/medicina56010015. 10.3390/medicina56010015 PMC7023392 31906017

[2] Bregenzer ME, Horst EN, Mehta P, Novak CM, Raghavan S, Snyder CS, et al. Integrated cancer tissue engineering models for precision medicine. PLoS One. 2019;14(5):e0216564. 10.1371/journal.pone.0216564. 10.1371/journal.pone.0216564 PMC6510431 31075118

[3] Luan YY, Dong N, Xie M, Xiao XZ, Yao YM (2014). The significance and regulatory mechanisms of innate immune cells in the development of sepsis. J Interf Cytokine Res.

[4] Pallmer K, Oxenius A (2016). Recognition and regulation of T cells by NK cells. Front Immunol.

[5] Srivastava A, Amreddy N, Pareek V, Chinnappan M, Ahmed R, Mehta M, et al. Progress in extracellular vesicle biology and their application in cancer medicine. Wiley Interdiscip Rev Nanomed Nanobiotechnol. 2020;12(4):e1621. 10.1002/wnan.1621. 10.1002/wnan.1621 PMC7317410 32131140

[6] Wang M, Yu F, Ding H, Wang Y, Li P, Wang K (2019). Emerging function and clinical values of exosomal microRNAs in cancer. Mol Ther Nucleic Acids.

[7] Javeed N, Mukhopadhyay D (2017). Exosomes and their role in the micro−/macro-environment: a comprehensive review. J Biomed Res.

[8] Azambuja JH, Ludwig N, Yerneni S, Rao A, Braganhol E, Whiteside TL (2020). Molecular profiles and immunomodulatory activities of glioblastoma-derived exosomes. Neurooncol Adv.

[9] Raposo G, Nijman HW, Stoorvogel W, Liejendekker R, Harding CV, Melief CJ, et al. B lymphocytes secrete antigen-presenting vesicles. J Exp Med. 1996;183(3):1161–72. 10.1084/jem.183.3.1161. 10.1084/jem.183.3.1161 PMC2192324 8642258

[10] Li Q, Wang H, Peng H, Huyan T, Cacalano NA. Exosomes: Versatile Nano Mediators of Immune Regulation. Cancers (Basel). 2019;11(10). 10.3390/cancers11101557. 10.3390/cancers11101557 PMC6826959 31615107

[11] Zitvogel L, Regnault A, Lozier A, Wolfers J, Flament C, Tenza D, et al. Eradication of established murine tumors using a novel cell-free vaccine: dendritic cell-derived exosomes. Nat Med. 1998;4(5):594–600. 10.1038/nm0598-594. 10.1038/nm0598-594 9585234

[12] Othman N, Jamal R, Abu N (2019). Cancer-derived exosomes as effectors of key inflammation- related players. Front Immunol.

[13] Bae S, Brumbaugh J, Bonavida B. Exosomes derived from cancerous and non-cancerous cells regulate the anti-tumor response in the tumor microenvironment. Genes Cancer. 2018;9(3–4):87–100. doi: 10.18632/genesandcancer.172. 10.18632/genesandcancer.172 PMC6086005 30108680

[14] Yan W, Jiang S (2020). Immune cell-derived exosomes in the cancer-immunity cycle. Trends Cancer.

[15] Xie F, Zhou X, Fang M, Li H, Su P, Tu Y, et al. Extracellular vesicles in cancer immune microenvironment and cancer immunotherapy. Adv Sci (Weinh). 2019;6(24):1901779. 10.1002/advs.201901779. 10.1002/advs.201901779 PMC6918121 31871860

[16] Hessvik NP, Llorente A (2018). Current knowledge on exosome biogenesis and release. Cell Mol Life Sci.

[17] Raposo G, Stoorvogel W (2013). Extracellular vesicles: exosomes, microvesicles, and friends. J Cell Biol.

[18] Tschuschke M, Kocherova I, Bryja A, Mozdziak P, Angelova Volponi A, Janowicz K, et al. Inclusion biogenesis, methods of isolation and clinical application of human cellular exosomes. J Clin Med. 2020;9(2). 10.3390/jcm9020436. 10.3390/jcm9020436 PMC7074492 32041096

[19] Alenquer M, Amorim MJ (2015). Exosome biogenesis, regulation, and function in viral infection. Viruses..

[20] Kalluri R (2016). The biology and function of exosomes in cancer. J Clin Invest.

[21] Tucci M, Mannavola F, Passarelli A, Stucci LS, Cives M, Silvestris F (2018). Exosomes in melanoma: a role in tumor progression, metastasis and impaired immune system activity. Oncotarget.

[22] Gurunathan S, Kang MH, Jeyaraj M, Qasim M, Kim JH. Review of the isolation, characterization, biological function, and multifarious therapeutic approaches of exosomes. Cells. 2019;8(4). doi: 10.3390/cells8040307. 10.3390/cells8040307 PMC6523673 30987213

[23] Vignard V, Labbe M, Marec N, Andre-Gregoire G, Jouand N, Fonteneau JF (2020). MicroRNAs in tumor Exosomes drive immune escape in melanoma. Cancer Immunol Res.

[24] Chaput N, Thery C (2011). Exosomes: immune properties and potential clinical implementations. Semin Immunopathol.

[25] Thery C, Zitvogel L, Amigorena S (2002). Exosomes: composition, biogenesis and function. Nat Rev Immunol..

[26] Pegtel DM, Gould SJ (2019). Exosomes. Annu Rev Biochem.

[27] Chettimada S, Lorenz DR, Misra V, Dillon ST, Reeves RK, Manickam C, et al. Exosome markers associated with immune activation and oxidative stress in HIV patients on antiretroviral therapy. Sci Rep. 2018;8(1):7227. 10.1038/s41598-018-25515-4. 10.1038/s41598-018-25515-4 PMC5940833 29740045

[28] Yang H, Sun L, Mao Y. The role of exosomes in tumor immunity. Ann Transl Med. 2018;6(Suppl 2):S116. 10.21037/atm.2018.12.03. 10.21037/atm.2018.12.03 PMC6330635 30740437

[29] Pardoll DM (2012). The blockade of immune checkpoints in cancer immunotherapy. Nat Rev Cancer.

[30] Grywalska E, Pasiarski M, Gozdz S, Rolinski J (2018). Immune-checkpoint inhibitors for combating T-cell dysfunction in cancer. Onco Targets Ther.

[31] Dong Y, Sun Q, Zhang X. PD-1 and its ligands are important immune checkpoints in cancer. Oncotarget. 2017;8(2):2171–86. doi: 10.18632/oncotarget.13895. 10.18632/oncotarget.13895 PMC5356790 27974689

[32] Ni L, Dong C (2017). New B7 family checkpoints in human cancers. Mol Cancer Ther.

[33] Xie F, Xu M, Lu J, Mao L, Wang S (2019). The role of exosomal PD-L1 in tumor progression and immunotherapy. Mol Cancer.

[34] Chen G, Huang AC, Zhang W, Zhang G, Wu M, Xu W (2018). Exosomal PD-L1 contributes to immunosuppression and is associated with anti-PD-1 response. Nature..

[35] Tang Y, Zhang P, Wang Y, Wang J, Su M, Wang Y (2020). The biogenesis, biology, and clinical significance of exosomal PD-L1 in cancer. Front Immunol..

[36] Olejarz W, Dominiak A, Zolnierzak A, Kubiak-Tomaszewska G, Lorenc T (2020). Tumor- derived exosomes in immunosuppression and immunotherapy. J Immunol Res.

[37] Cordonnier M, Nardin C, Chanteloup G, Derangere V, Algros MP, Arnould L (2020). Tracking the evolution of circulating exosomal-PD-L1 to monitor melanoma patients. J Extracell Vesicles..

[38] Song Y, Wu L, Yang C (2018). Exosomal PD-L1: an effective liquid biopsy target to predict immunotherapy response. National Science Review.

[39] Gunasekaran M, Russo A, Cardona AF, de Miguel PD, Lapidus R, Cooper B, et al. Exosomal PD-L1 expression as non-invasive biomarker for immune checkpoint inhibitors in non-small cell lung cancer. J Immunol. 2020;204(1 Supplement):90.10–0.

[40] Ruffner MA, Kim SE, Bianco NR, Francisco LM, Sharpe AH, Robbins PD (2009). B7-1/2, but not PD-L1/2 molecules, are required on IL-10-treated tolerogenic DC and DC-derived exosomes for in vivo function. Eur J Immunol.

[41] Zhou K, Guo S, Li F, Sun Q, Liang G (2020). Exosomal PD-L1: new insights into tumor immune escape mechanisms and therapeutic strategies. Front Cell Dev Biol..

[42] Knox MC, Ni J, Bece A, Bucci J, Chin Y, Graham PH (2020). A Clinician’s guide to cancer- derived exosomes: immune interactions and therapeutic implications. Front Immunol..

[43] Haraszti RA, Didiot MC, Sapp E, Leszyk J, Shaffer SA, Rockwell HE (2016). High- resolution proteomic and lipidomic analysis of exosomes and microvesicles from different cell sources. J Extracell Vesicles..

[44] Skotland T, Sagini K, Sandvig K, Llorente A (2020). An emerging focus on lipids in extracellular vesicles. Adv Drug Deliv Rev.

[45] Skotland T, Sandvig K, Llorente A (2017). Lipids in exosomes: current knowledge and the way forward. Prog Lipid Res.

[46] Skotland T, Ekroos K, Kauhanen D, Simolin H, Seierstad T, Berge V (2017). Molecular lipid species in urinary exosomes as potential prostate cancer biomarkers. Eur J Cancer..

[47] Hough KP, Wilson LS, Trevor JL, Strenkowski JG, Maina N, Kim YI (2018). Unique lipid signatures of extracellular vesicles from the airways of asthmatics. Sci Rep..

[48] Brzozowski JS, Jankowski H, Bond DR, McCague SB, Munro BR, Predebon MJ (2018). Lipidomic profiling of extracellular vesicles derived from prostate and prostate cancer cell lines. Lipids Health Dis.

[49] Cheng L, Zhang K, Qing Y, Li D, Cui M, Jin P (2020). Proteomic and lipidomic analysis of exosomes derived from ovarian cancer cells and ovarian surface epithelial cells. J Ovarian Res..

[50] Cheng J, Meng J, Zhu L, Peng Y (2020). Exosomal noncoding RNAs in glioma: biological functions and potential clinical applications. Mol Cancer.

[51] Zhou X, Xie F, Wang L, Zhang L, Zhang S, Fang M (2020). The function and clinical application of extracellular vesicles in innate immune regulation. Cell Mol Immunol..

[52] Mills J, Capece M, Cocucci E, Tessari A, Palmieri D. Cancer-derived extracellular vesicle-associated microRNAs in intercellular communication: one cell’s trash is another cell’s treasure. Int J Mol Sci. 2019;20(24). 10.3390/ijms20246109. 10.3390/ijms20246109 PMC6940802 31817101

[53] Turchinovich A, Drapkina O, Tonevitsky A (2019). Transcriptome of extracellular vesicles: state-of-the-art. Front Immunol.

[54] Pezzuto F, Buonaguro L, Buonaguro FM, Tornesello ML. The role of circulating free dna and microRNA in non-invasive diagnosis of HBV- and HCV-related hepatocellular carcinoma. Int J Mol Sci. 2018;19(4). doi: 10.3390/ijms19041007. 10.3390/ijms19041007 PMC5979406 29597259

[55] Jeppesen DK, Fenix AM, Franklin JL, Higginbotham JN, Zhang Q, Zimmerman LJ, Liebler DC, Ping J, Liu Q, Evans R, Fissell WH, Patton JG, Rome LH, Burnette DT, Coffey RJ Reassessment of exosome composition. Cell. 2019;177(2):428–445 e18. doi: 10.1016/j.cell.2019.02.029. 10.1016/j.cell.2019.02.029 PMC6664447 30951670

[56] Halvaei S, Daryani S, Eslami SZ, Samadi T, Jafarbeik-Iravani N, Bakhshayesh TO (2018). Exosomes in cancer liquid biopsy: a focus on breast cancer. Mol Ther Nucleic Acids..

[57] Tai YL, Chu PY, Lee BH, Chen KC, Yang CY, Kuo WH (2019). Basics and applications of tumor-derived extracellular vesicles. J Biomed Sci..

[58] Yanez-Mo M, Siljander PR, Andreu Z, Zavec AB, Borras FE, Buzas EI (2015). Biological properties of extracellular vesicles and their physiological functions. J Extracell Vesicles..

[59] Schwarzenbach H, Gahan PB. MicroRNA shuttle from cell-to-cell by exosomes and its impact in cancer. Noncoding RNA. 2019;5(1). 10.3390/ncrna5010028. 10.3390/ncrna5010028 PMC6468647 30901915

[60] Bhome R, Del Vecchio F, Lee GH, Bullock MD, Primrose JN, Sayan AE (2018). Exosomal microRNAs (exomiRs): small molecules with a big role in cancer. Cancer Lett.

[61] Fabbri M, Paone A, Calore F, Galli R, Croce CM (2013). A new role for microRNAs, as ligands of Toll-like receptors. RNA Biol.

[62] Yang M, Chen J, Su F, Yu B, Su F, Lin L (2011). Microvesicles secreted by macrophages shuttle invasion-potentiating microRNAs into breast cancer cells. Mol Cancer..

[63] Berchem G, Noman MZ, Bosseler M, Paggetti J, Baconnais S, Le Cam E (2016). Hypoxic tumor-derived microvesicles negatively regulate NK cell function by a mechanism involving TGF-beta and miR23a transfer. Oncoimmunology..

[64] Ding G, Zhou L, Qian Y, Fu M, Chen J, Chen J, et al. Pancreatic cancer-derived exosomes transfer miRNAs to dendritic cells and inhibit RFXAP expression via miR-212-3p. Oncotarget. 2015;6(30):29877–88. 10.18632/oncotarget.4924. 10.18632/oncotarget.4924 PMC4745769 26337469

[65] Zhou M, Chen J, Zhou L, Chen W, Ding G, Cao L (2014). Pancreatic cancer derived exosomes regulate the expression of TLR4 in dendritic cells via miR-203. Cell Immunol.

[66] Ye SB, Zhang H, Cai TT, Liu YN, Ni JJ, He J (2016). Exosomal miR-24-3p impedes T-cell function by targeting FGF11 and serves as a potential prognostic biomarker for nasopharyngeal carcinoma. J Pathol..

[67] Cheng Z, Ma R, Tan W, Zhang L (2014). MiR-152 suppresses the proliferation and invasion of NSCLC cells by inhibiting FGF2. Exp Mol Med.

[68] Alfonsi R, Grassi L, Signore M, Bonci D. The double face of exosome-carried microRNAs in cancer immunomodulation. Int J Mol Sci. 2018;19(4). 10.3390/ijms19041183. 10.3390/ijms19041183 PMC5979514 29652798

[69] Nakano T, Chen IH, Wang CC, Chen PJ, Tseng HP, Huang KT (2019). Circulating exosomal miR-92b: its role for cancer immunoediting and clinical value for prediction of posttransplant hepatocellular carcinoma recurrence. Am J Transplant..

[70] Ying X, Wu Q, Wu X, Zhu Q, Wang X, Jiang L, et al. Epithelial ovarian cancer-secreted exosomal miR-222-3p induces polarization of tumor-associated macrophages. Oncotarget. 2016;7(28):43076–87. 10.18632/oncotarget.9246. 10.18632/oncotarget.9246 PMC5190009 27172798

[71] Czernek L, Duchler M (2017). Functions of cancer-derived extracellular vesicles in immunosuppression. Arch Immunol Ther Exp (Warsz).

[72] Su MW, Yu SL, Lin WC, Tsai CH, Chen PH, Lee YL (2016). Smoking-related microRNAs and mRNAs in human peripheral blood mononuclear cells. Toxicol Appl Pharmacol.

[73] Yin Y, Cai X, Chen X, Liang H, Zhang Y, Li J (2014). Tumor-secreted miR-214 induces regulatory T cells: a major link between immune evasion and tumor growth. Cell Res..

[74] Nowak M, Klink M. The role of tumor-associated macrophages in the progression and chemoresistance of ovarian cancer. Cells. 2020;9(5). 10.3390/cells9051299. 10.3390/cells9051299 PMC7290435 32456078

[75] Cooks T, Pateras IS, Jenkins LM, Patel KM, Robles AI, Morris J (2018). Mutant p53 cancers reprogram macrophages to tumor supporting macrophages via exosomal miR-1246. Nat Commun..

[76] Eichmuller SB, Osen W, Mandelboim O, Seliger B. Immune modulatory microRNAs involved in tumor attack and tumor immune escape. J Natl Cancer Inst. 2017;109(10). 10.1093/jnci/djx034. 10.1093/jnci/djx034 28383653

[77] Jia L, Xi Q, Wang H, Zhang Z, Liu H, Cheng Y (2017). miR-142-5p regulates tumor cell PD-L1 expression and enhances anti-tumor immunity. Biochem Biophys Res Commun..

[78] Jablonska J, Pietrowska M, Ludwig S, Lang S, Thakur BK. Challenges in the isolation and proteomic analysis of cancer exosomes-implications for translational research. Proteomes. 2019;7(2). 10.3390/proteomes7020022. 10.3390/proteomes7020022 PMC6631388 31096692

[79] Fernando MR, Jiang C, Krzyzanowski GD, Ryan WL (2017). New evidence that a large proportion of human blood plasma cell-free DNA is localized in exosomes. PLoS One.

[80] Guescini M, Guidolin D, Vallorani L, Casadei L, Gioacchini AM, Tibollo P (2010). C2C12 myoblasts release micro-vesicles containing mtDNA and proteins involved in signal transduction. Exp Cell Res..

[81] Thakur BK, Zhang H, Becker A, Matei I, Huang Y, Costa-Silva B (2014). Double-stranded DNA in exosomes: a novel biomarker in cancer detection. Cell Res..

[82] Balaj L, Lessard R, Dai L, Cho YJ, Pomeroy SL, Breakefield XO (2011). Tumour microvesicles contain retrotransposon elements and amplified oncogene sequences. Nat Commun..

[83] Taghikhani A, Farzaneh F, Sharifzad F, Mardpour S, Ebrahimi M, Hassan ZM (2020). Engineered tumor-derived extracellular vesicles: potentials in cancer immunotherapy. Front Immunol.

[84] Kurywchak P, Tavormina J, Kalluri R (2018). The emerging roles of exosomes in the modulation of immune responses in cancer. Genome Med.

[85] Lian Q, Xu J, Yan S, Huang M, Ding H, Sun X (2017). Chemotherapy-induced intestinal inflammatory responses are mediated by exosome secretion of double-strand DNA via AIM2 inflammasome activation. Cell Res..

[86] Kitai Y, Kawasaki T, Sueyoshi T, Kobiyama K, Ishii KJ, Zou J (2017). DNA-containing exosomes derived from cancer cells treated with topotecan activate a STING- dependent pathway and reinforce antitumor immunity. J Immunol..

[87] Maia J, Caja S, Strano Moraes MC, Couto N, Costa-Silva B (2018). Exosome-based cell-cell communication in the tumor microenvironment. Front Cell Dev Biol..

[88] Maheshwari S, Singh AK, Arya RK, Pandey D, Singh A, Datta D. Exosomes: emerging players of intercellular communication in tumor microenvironment. Discoveries (Craiova). 2014;2(3):e26. 10.15190/d.2014.18. 10.15190/d.2014.18 PMC6941565 32309554

[89] Gao D, Jiang L (2018). Exosomes in cancer therapy: a novel experimental strategy. Am J Cancer Res.

[90] Lindenbergh MFS, Stoorvogel W (2018). Antigen presentation by extracellular vesicles from professional antigen-presenting cells. Annu Rev Immunol.

[91] Robbins PD, Morelli AE (2014). Regulation of immune responses by extracellular vesicles. Nat Rev Immunol.

[92] Lu M, Huang B, Hanash SM, Onuchic JN, Ben-Jacob E (2014). Modeling putative therapeutic implications of exosome exchange between tumor and immune cells. Proc Natl Acad Sci U S A.

[93] Ning Y, Shen K, Wu Q, Sun X, Bai Y, Xie Y (2018). Tumor exosomes block dendritic cells maturation to decrease the T cell immune response. Immunol Lett..

[94] Shen Y, Guo D, Weng L, Wang S, Ma Z, Yang Y (2017). Tumor-derived exosomes educate dendritic cells to promote tumor metastasis via HSP72/HSP105-TLR2/TLR4 pathway. Oncoimmunology..

[95] Batista IA, Melo SA. Exosomes and the future of immunotherapy in pancreatic cancer. Int J Mol Sci. 2019;20(3). 10.3390/ijms20030567. 10.3390/ijms20030567 PMC6387297 30699928

[96] Tung SL, Fanelli G, Matthews RI, Bazoer J, Letizia M, Vizcay-Barrena G (2020). Regulatory T cell extracellular vesicles modify T-effector cell cytokine production and protect against human skin allograft damage. Front Cell Dev Biol..

[97] Tung SL, Boardman DA, Sen M, Letizia M, Peng Q, Cianci N (2018). Regulatory T cell- derived extracellular vesicles modify dendritic cell function. Sci Rep..

[98] Cheng H, Wang Z, Fu L, Xu T (2019). Macrophage polarization in the development and progression of ovarian cancers: an overview. Front Oncol.

[99] Baig MS, Roy A, Rajpoot S, Liu D, Savai R, Banerjee S (2020). Tumor-derived exosomes in the regulation of macrophage polarization. Inflamm Res..

[100] Smith HA, Kang Y (2013). The metastasis-promoting roles of tumor-associated immune cells. J Mol Med (Berl).

[101] Mantovani A, Marchesi F, Malesci A, Laghi L, Allavena P (2017). Tumour-associated macrophages as treatment targets in oncology. Nat Rev Clin Oncol.

[102] Yang M, McKay D, Pollard JW, Lewis CE (2018). Diverse functions of macrophages in different tumor microenvironments. Cancer Res.

[103] Ran S, Montgomery KE (2012). Macrophage-mediated lymphangiogenesis: the emerging role of macrophages as lymphatic endothelial progenitors. Cancers (Basel).

[104] Meng W, Hao Y, He C, Li L, Zhu G (2019). Exosome-orchestrated hypoxic tumor microenvironment. Mol Cancer.

[105] Zhou J, Li X, Wu X, Zhang T, Zhu Q, Wang X (2018). Exosomes released from tumor- associated macrophages transfer miRNAs that induce a Treg/Th17 cell imbalance in epithelial ovarian cancer. Cancer Immunol Res..

[106] Blanchard N, Lankar D, Faure F, Regnault A, Dumont C, Raposo G (2002). TCR activation of human T cells induces the production of exosomes bearing the TCR/CD3/zeta complex. J Immunol..

[107] Wahlgren J, Karlson Tde L, Glader P, Telemo E, Valadi H (2012). Activated human T cells secrete exosomes that participate in IL-2 mediated immune response signaling. PLoS One.

[108] Zhang L, Yu D (2019). Exosomes in cancer development, metastasis, and immunity. Biochim Biophys Acta Rev Cancer.

[109] Cai Z, Yang F, Yu L, Yu Z, Jiang L, Wang Q (2012). Activated T cell exosomes promote tumor invasion via Fas signaling pathway. J Immunol..

[110] Srivastava A, Babu A, Filant J, Moxley KM, Ruskin R, Dhanasekaran D (2016). Exploitation of exosomes as Nanocarriers for gene-, chemo-, and immune-therapy of cancer. J Biomed Nanotechnol..

[111] Kim MS, Haney MJ, Zhao Y, Mahajan V, Deygen I, Klyachko NL (2016). Development of exosome-encapsulated paclitaxel to overcome MDR in cancer cells. Nanomedicine..

[112] Yong T, Zhang X, Bie N, Zhang H, Zhang X, Li F (2019). Tumor exosome-based nanoparticles are efficient drug carriers for chemotherapy. Nat Commun..

[113] Srivastava A, Amreddy N, Razaq M, Towner R, Zhao YD, Ahmed RA (2018). Exosomes as theranostics for lung cancer. Adv Cancer Res.

[114] Srivastava A, Moxley K, Ruskin R, Dhanasekaran DN, Zhao YD, Ramesh R (2018). A non- invasive liquid biopsy screening of urine-derived exosomes for miRNAs as biomarkers in endometrial cancer patients. AAPS J..

[115] Chinnappan M, Srivastava A, Amreddy N, Razaq M, Pareek V, Ahmed R (2020). Exosomes as drug delivery vehicle and contributor of resistance to anticancer drugs. Cancer Lett..

[116] Tan A, Rajadas J, Seifalian AM (2013). Exosomes as nano-theranostic delivery platforms for gene therapy. Adv Drug Deliv Rev.

[117] He C, Zheng S, Luo Y, Wang B (2018). Exosome theranostics: biology and translational medicine. Theranostics..

[118] Zhan Q, Yi K, Qi H, Li S, Li X, Wang Q (2020). Engineering blood exosomes for tumor- targeting efficient gene/chemo combination therapy. Theranostics..

[119] Tian Y, Li S, Song J, Ji T, Zhu M, Anderson GJ (2014). A doxorubicin delivery platform using engineered natural membrane vesicle exosomes for targeted tumor therapy. Biomaterials..

[120] Kamerkar S, LeBleu VS, Sugimoto H, Yang S, Ruivo CF, Melo SA (2017). Exosomes facilitate therapeutic targeting of oncogenic KRAS in pancreatic cancer. Nature..

[121] Naseri M, Bozorgmehr M, Zoller M, Ranaei Pirmardan E, Madjd Z (2020). Tumor-derived exosomes: the next generation of promising cell-free vaccines in cancer immunotherapy. Oncoimmunology..

[122] Nikfarjam S, Rezaie J, Kashanchi F, Jafari R (2020). Dexosomes as a cell-free vaccine for cancer immunotherapy. J Exp Clin Cancer Res..

[123] Hiltbrunner S, Larssen P, Eldh M, Martinez-Bravo MJ, Wagner AK, Karlsson MC, et al. Exosomal cancer immunotherapy is independent of MHC molecules on exosomes. Oncotarget. 2016;7(25):38707–17. 10.18632/oncotarget.9585. 10.18632/oncotarget.9585 PMC5122422 27231849

[124] Forsberg MH, Kink JA, Hematti P, Capitini CM (2020). Mesenchymal stromal cells and exosomes: progress and challenges. Front Cell Dev Biol.

[125] Cheng Y, Zeng Q, Han Q, Xia W (2019). Effect of pH, temperature and freezing-thawing on quantity changes and cellular uptake of exosomes. Protein Cell.

[126] Jeyaram A, Jay SM (2017). Preservation and storage stability of extracellular vesicles for therapeutic applications. AAPS J.

[127] Reclusa P, Taverna S, Pucci M, Durendez E, Calabuig S, Manca P, et al. Exosomes as diagnostic and predictive biomarkers in lung cancer. J Thorac Dis. 2017;9(Suppl 13):S1373-S82. 10.21037/jtd.2017.10.67. 10.21037/jtd.2017.10.67 PMC5676107 29184676

[128] Revelo AE, Martin A, Velasquez R, Kulandaisamy PC, Bustamante J, Keshishyan S, et al. Liquid biopsy for lung cancers: an update on recent developments. Ann Transl Med. 2019;7(15):349. doi: 10.21037/atm.2019.03.28. 10.21037/atm.2019.03.28 PMC6712255 31516895

[129] Rodriguez M, Silva J, Lopez-Alfonso A, Lopez-Muniz MB, Pena C, Dominguez G (2014). Different exosome cargo from plasma/bronchoalveolar lavage in non-small-cell lung cancer. Genes Chromosomes Cancer.

[130] Lin J, Wang Y, Zou YQ, Chen X, Huang B, Liu J (2016). Differential miRNA expression in pleural effusions derived from extracellular vesicles of patients with lung cancer, pulmonary tuberculosis, or pneumonia. Tumour Biol..

[131] Hydbring P, De Petris L, Zhang Y, Branden E, Koyi H, Novak M (2018). Exosomal RNA- profiling of pleural effusions identifies adenocarcinoma patients through elevated miR-200 and LCN2 expression. Lung Cancer.

[132] Li Y, Zhang Y, Qiu F, Qiu Z (2011). Proteomic identification of exosomal LRG1: a potential urinary biomarker for detecting NSCLC. Electrophoresis..

[133] Lin SY, Chang CH, Wu HC, Lin CC, Chang KP, Yang CR (2016). Proteome profiling of urinary exosomes identifies alpha 1-antitrypsin and H2B1K as diagnostic and prognostic biomarkers for urothelial carcinoma. Sci Rep..

[134] Long JD, Sullivan TB, Humphrey J, Logvinenko T, Summerhayes KA, Kozinn S (2015). A non-invasive miRNA based assay to detect bladder cancer in cell-free urine. Am J Transl Res..

[135] Fujita K, Nonomura N (2018). Urinary biomarkers of prostate cancer. Int J Urol.

[136] McKiernan J, Donovan MJ, O’Neill V, Bentink S, Noerholm M, Belzer S (2016). A novel urine exosome gene expression assay to predict high-grade prostate cancer at initial biopsy. JAMA Oncol.

[137] Overbye A, Skotland T, Koehler CJ, Thiede B, Seierstad T, Berge V, et al. Identification of prostate cancer biomarkers in urinary exosomes. Oncotarget. 2015;6(30):30357–76. 10.18632/oncotarget.4851. 10.18632/oncotarget.4851 PMC4745805 26196085

[138] Rodriguez M, Bajo-Santos C, Hessvik NP, Lorenz S, Fromm B, Berge V (2017). Identification of non-invasive miRNAs biomarkers for prostate cancer by deep sequencing analysis of urinary exosomes. Mol Cancer.

[139] Raimondo F, Morosi L, Corbetta S, Chinello C, Brambilla P, Della Mina P (2013). Differential protein profiling of renal cell carcinoma urinary exosomes. Mol BioSyst..

[140] Sauter ER, Reidy D (2017). How exosomes in human breast milk may influence breast cancer risk. Transl Cancer Res.

[141] Wakefield LM, Roberts AB (2002). TGF-beta signaling: positive and negative effects on tumorigenesis. Curr Opin Genet Dev.

[142] Rontogianni S, Synadaki E, Li B, Liefaard MC, Lips EH, Wesseling J (2019). Proteomic profiling of extracellular vesicles allows for human breast cancer subtyping. Commun Biol..

[143] Wang Z, Li F, Rufo J, Chen C, Yang S, Li L (2020). Acoustofluidic salivary exosome isolation: a liquid biopsy compatible approach for human papillomavirus-associated oropharyngeal cancer detection. J Mol Diagn..

[144] Nonaka T, Wong DTW (2017). Saliva-Exosomics in cancer: molecular characterization of cancer-derived exosomes in saliva. Enzymes..

[145] Perez-Callejo D, Romero A, Provencio M, Torrente M. Liquid biopsy based biomarkers in non-small cell lung cancer for diagnosis and treatment monitoring. Transl Lung Cancer Res. 2016;5(5):455–65. 10.21037/tlcr.2016.10.07. 10.21037/tlcr.2016.10.07 PMC5099509 27826527

[146] De Rubis G, Rajeev Krishnan S, Bebawy M (2019). Liquid biopsies in cancer diagnosis, monitoring, and prognosis. Trends Pharmacol Sci.

[147] Li X, Corbett AL, Taatizadeh E, Tasnim N, Little JP, Garnis C (2019). Challenges and opportunities in exosome research-perspectives from biology, engineering, and cancer therapy. APL Bioeng..

[148] Ludwig N, Whiteside TL, Reichert TE. Challenges in exosome isolation and analysis in health and disease. Int J Mol Sci. 2019;20(19). 10.3390/ijms20194684. 10.3390/ijms20194684 PMC6801453 31546622

